# Depression-Related Nutritional Risk and Physical Performance in Middle- to Older-Aged Adults: Cross-Sectional Secondary Analysis of Tree-Based and Regression Approaches

**DOI:** 10.2196/94510

**Published:** 2026-06-01

**Authors:** Yi-Chen Wu, Shu-Fen Shen, Fong-Ping Tang, Chia-Te Chen, Liang-Kung Chen, Kuan-Yu Peng, Heng-Hsin Tung

**Affiliations:** 1 Graduate Institute of Clinical Nursing, College of Medicine, National Chung Hsing University, Taichung, Taiwan; 2 Department of Nursing, Mackay Medical University, Taipei, Taiwan; 3 Institute of Clinical Nursing, National Yang Ming Chiao Tung University, Taipei, Taiwan; 4 Department of Nursing, Taipei Municipal Gan-Dau Hospital, Taipei, Taiwan; 5 Taipei Veterans General Hospital, Taipei, Taiwan; 6 Center for Healthy Longevity and Aging Sciences, National Yang Ming Chiao Tung University, Taipei, Taiwan; 7 Taiwan Semiconductor Manufacturing Company (Taiwan), Hsin Chu, Taiwan; 8 College of Nursing, National Yang Ming Chiao Tung University, No. 155, Sec. 2, Linong St, Beitou District, Taipei, 112304, Taiwan, 886-2-2827-5657

**Keywords:** depression, nutritional status, physical functional performance, machine learning, decision trees, risk stratification, aging, geriatric assessment

## Abstract

**Background:**

Depressive symptoms are linked to nutritional vulnerability and functional decline in aging populations, but their relationships with nutritional risk and lower-extremity physical performance are often examined separately. Tree-based exploratory approaches may provide transparent subgroup characterization.

**Objective:**

This study aimed to examine the cross-sectional associations of depressive symptoms with nutritional risk and lower-extremity physical performance among community-dwelling middle- to older-aged adults and to compare a tree-based exploratory approach with regression-based methods for characterizing depression-related patterns within the study sample.

**Methods:**

This cross-sectional secondary analysis included 1010 community-dwelling adults aged ≥50 years recruited from 1 hospital and 3 community centers in northern Taiwan. Depressive symptoms were assessed using the Geriatric Depression Scale–Short Form (GDS-15). Nutritional status was measured using the Mini Nutritional Assessment–Short Form (MNA-SF), and physical performance was evaluated using the Short Physical Performance Battery (SPPB), gait speed, and the timed up and go (TUG) test. Sociodemographic, lifestyle, and clinical variables were included as covariates. Outcomes were dichotomized using established clinical cutoffs. *χ*² automatic interaction detection (CHAID) decision trees were used as a tree-based exploratory approach for subgroup characterization. Multivariable logistic regression and least absolute shrinkage and selection operator (LASSO) logistic regression models were used for comparison. Model performance was evaluated using the area under the receiver operating characteristic curve (AUC), sensitivity, specificity, and classification accuracy.

**Results:**

Of the 1010 participants, 143 (14.2%) screened positive for depressive symptoms (GDS-15 ≥5). Compared with participants without depressive symptoms, those with depressive symptoms had poorer nutritional status and lower-extremity physical performance, including a higher prevalence of nutritional risk (53/143, 37.1% vs 105/867, 12.1%; *P*<.001), SPPB impairment (53/143, 37.1% vs 118/867, 13.6%; *P*<.001), gait speed impairment (73/143, 51% vs 270/865, 31.2%; *P*<.001), and TUG impairment (25/143, 17.5% vs 55/864, 6.4%; *P*<.001). Across CHAID models, GDS-15 score was consistently selected as the primary splitting variable, while BMI, calf circumference, age, education level, and comorbidity severity provided additional hierarchical refinement of subgroup patterns. In comparative model analyses, LASSO logistic regression analysis had the highest classification performance for the MNA-SF, SPPB, and gait speed outcomes, whereas CHAID provided transparent, rule-based subgroup characterization with acceptable within-sample classification performance. Model performance for TUG was less consistent across approaches.

**Conclusions:**

In this community-based sample of adults aged 50 years and older, depressive symptom severity was associated with nutritional vulnerability and poorer lower-extremity physical performance. CHAID identified hierarchical subgroup patterns linking depressive symptoms, including subthreshold levels, with nutritional and functional vulnerability. Although LASSO logistic regression analysis had higher classification performance, CHAID offered transparent, rule-based subgroup characterization. These findings support the relevance of integrating depression screening with nutritional and functional assessment in prevention-oriented geriatric care, although further validation is needed.

## Introduction

Depression, nutritional risk, and lower-extremity function are key domains within comprehensive geriatric assessment, as they reflect important medical, psychosocial, and functional dimensions of health, and are routinely considered in geriatric care due to their high prevalence, close interrelationships, and strong associations with disability, frailty, and reduced quality of life [1]. Depression is a common mental health condition and a major public health concern in this population. Depressive symptoms are particularly prevalent among adults aged 50 years and older and have been associated with nutritional vulnerability, accelerated functional decline, and increased premature mortality [2-5]. Malnutrition risk is also common during the transition from midlife to older adulthood, a period often characterized by multimorbidity, retirement-related adjustment, and changing social roles that may weaken psychological resilience and overall health status [6-8]. In addition, greater depressive symptom burden has been shown to have dose-response associations with adverse health outcomes in later life [9]. Decline in lower-extremity physical performance, reflected in slower gait speed and reduced muscle strength, is likewise strongly associated with disability, falls, and progression to frailty among older adults [10-12].

Understanding the mechanisms underlying these associations is important for clinical assessment and intervention. Depressive symptoms may contribute to functional decline through several pathways, including reduced motivation, appetite changes, persistent fatigue, and lower participation in physical and daily activities [8,10,13-15]. These changes may, in turn, accelerate mobility decline and increase vulnerability to social withdrawal and reduced independence. Nutritional status is another important and modifiable determinant of health in aging populations [16]. Poor nutritional intake may lead to loss of muscle mass, sarcopenia, and frailty [15,17], which can further impair gait, balance, and overall physical function [11,18]. From a clinical perspective, depressive symptoms and nutritional vulnerability are both highly relevant because they are potentially modifiable and closely related to mobility outcomes. Together, depression, malnutrition, and impaired physical performance may reinforce one another over time [8,11,19], creating a downward spiral that contributes to disability, frailty, and increased mortality risk [12,13].

Although previous studies have documented associations between depressive symptoms and nutritional risk, depressive symptoms and mobility decline, and nutritional risk and physical performance, these relationships have often been examined separately rather than within an integrated framework in a single community-based sample [13,14,19-21]. Conventional regression approaches have been widely used to characterize such associations, but they may be less well suited to identifying potential nonlinear patterns, interaction structures, and clinically relevant thresholds across multiple correlated factors [22]. In this context, a selected tree-based analytic approach, *χ*² automatic interaction detection (CHAID), may provide complementary value by yielding visually explicit subgroup splits and transparent rule-based patterns for exploratory subgroup characterization [22-25]. In this study, CHAID was used as a transparent exploratory analytic tool rather than as a comprehensive representation of modern interpretable machine learning. However, few studies have applied both a tree-based exploratory approach and conventional regression methods to examine depression-related patterns in nutritional vulnerability and lower-extremity physical performance among community-dwelling middle- to older-aged adults.

Given these considerations, integrating a tree-based exploratory approach with conventional regression methods may provide a more comprehensive understanding of how depressive symptoms relate to nutritional risk and lower-extremity physical performance in community-dwelling middle- to older-aged adults. This study had 2 aims: (1) to examine the cross-sectional associations between depressive symptoms and 2 clinically relevant outcomes, nutritional risk and lower-extremity physical performance, among community-dwelling middle- to older-aged adults and (2) to compare a tree-based exploratory approach with conventional regression methods for characterizing depression-related patterns within the study sample. We hypothesized that greater depressive symptom severity would be associated with higher nutritional risk and poorer lower-extremity physical performance and that the tree-based exploratory approach would provide complementary pattern-based insights beyond regression estimates alone.

## Methods

### Study Design and Population

We conducted a cross-sectional secondary analysis using data from the Gan Dau Healthy Longevity Plan in Taiwan to examine the associations of depressive symptoms with nutritional risk and lower-extremity physical performance among community-dwelling middle- to older-aged adults. A total of 1010 participants aged 50 years or older were recruited through convenience sampling from 1 hospital and 3 community centers in northern Taiwan between January 2024 and September 2024. Participants were eligible if they were able to communicate in Mandarin or Taiwanese and were able to provide informed consent.

All assessments were completed by trained personnel after written informed consent was obtained. Standardized assessment procedures were used to reduce measurement bias, and all analytic data were fully deidentified to protect participant confidentiality. Sample size was determined by the number of eligible participants in the parent study, and no a priori power calculation was performed. Because participants were recruited through convenience sampling from 1 hospital and 3 community centers, selection bias may have occurred, and the enrolled participants may have been healthier or more motivated than nonparticipants.

Depressive symptoms were assessed using the 15-item Geriatric Depression Scale–Short Form (GDS-15). Nutritional status was assessed using the Mini Nutritional Assessment–Short Form (MNA-SF). Lower-extremity physical performance was assessed using the Short Physical Performance Battery (SPPB), gait speed, and the timed up-and-go (TUG) test. In this analysis, depressive symptom severity was treated as the main explanatory variable, whereas nutritional risk and lower-extremity physical performance were treated as study outcomes. This study was reported in accordance with the STROBE (Strengthening the Reporting of Observational Studies in Epidemiology) guidelines.

### Ethical Considerations

This study was approved by the Institutional Review Board of Taipei Veterans General Hospital, Taiwan (number 2023-10-001B#1). Written informed consent was obtained from all participants or legally authorized representatives, when necessary, in accordance with the Declaration of Helsinki.

Participant privacy was protected by collecting data anonymously and analyzing the data in aggregate form. No personally identifiable information was reported. The data were stored securely and were accessible only to the research team. Participants did not receive any compensation for participating in this study.

### Measures

#### Depressive Symptoms

Depressive symptoms were assessed using the GDS-15, a validated screening tool widely used in older populations [26]. The abbreviated version of the 30-item GDS was developed by Yesavage and colleagues [27], and the Mandarin Chinese version was culturally adapted by Yeh and colleagues [28]. Each item is answered with Yes or No; total scores range from 0 to 15. A cutoff of ≥5 indicates possible depressive symptoms [29,30].

#### Nutritional Status

Nutritional status was measured using the MNA-SF, a 6-item tool assessing food intake, weight loss, mobility, psychological stress, neuropsychological problems, and BMI or calf circumference [31,32]. Total scores (0‐14) classify individuals as well-nourished (12-14), at risk (8-11), or malnourished (≤7). The MNA-SF demonstrates strong validity and predictive accuracy in older populations [32-34].

#### Lower-Extremity Physical Performance

Lower-extremity physical performance was assessed using the SPPB, gait speed, and TUG. The SPPB includes balance tests, a 4-meter usual-pace walk, and a 5-time chair stand, each scored from 0 to 4 for a total score of 0 to 12 [35-37]. Gait speed was measured over 6 meters and expressed in meters per second (m/s) [36]. The TUG evaluates mobility by timing the sequence of standing up, walking 10 feet, turning, returning, and sitting down [38,39].

### Covariates

Covariates included demographics (age, sex, marital status, education, living arrangement, economic status), health behaviors (regular exercise, leisure activity, fall history), anthropometrics (BMI, abdominal and calf circumferences), and multimorbidity. Multimorbidity was measured using the Cumulative Illness Rating Scale for Geriatrics (CIRS-G), developed by Linn et al [40] and adapted for older adult populations by Miller et al [41] with a 14-system scoring manual [42]. The CIRS-G total score reflects overall comorbidity burden, and the Severity Index (CIRS-G SI) represents the average severity across organ systems [42,43].

### Statistical Analysis

Categorical variables were summarized as n (%), and continuous variables were summarized as median and IQR. Group differences by depression status (GDS-15 score <5 vs ≥5) were examined using the Mann-Whitney *U* test for continuous variables and the *χ*² test or Fisher exact test for categorical variables, as appropriate, with a 2-sided α of .05. Nutritional status was dichotomized as well-nourished (MNA-SF≥12) versus at risk or malnourished (MNA-SF≤11). Impairment thresholds for lower-extremity performance followed established criteria: SPPB≤9, gait speed≤1.0 m/s (6-m walk), and TUG≥12 seconds [38,39]. These binary outcomes were used in all analytic models, including CHAID, logistic regression, and least absolute shrinkage and selection operator (LASSO) logistic regression. Because missingness was <5% for all variables, complete-case analysis was applied; given the low proportion of missing data, the potential impact on the results was considered limited.

The primary exploratory analytic approach used CHAID to identify data-driven thresholds and nonlinear interaction patterns related to impaired nutritional status and lower-extremity physical performance [44,45]. Outcomes were analyzed as binary variables based on the aforementioned clinical cutoffs. Candidate predictors included GDS-15 score, sociodemographic characteristics, lifestyle behaviors, anthropometric measures, and multimorbidity indicators. Predictors were retained in their original scales, and CHAID generated multiway splits using Bonferroni-adjusted *χ*² tests (*α*=.05). Tree growth was constrained to a maximum depth of 3 levels, with minimum parent and child node sizes of 100 and 50, respectively. For each terminal node, we summarized sample size, class distribution, and misclassification risk. Model evaluation was based on an internal 80% training set and a 20% testing set [46]. Classification accuracy was calculated in both datasets, and the applicability of the training set tree structure to the test set was examined descriptively. Because no cross-validation, bootstrapping, nor external validation was performed, the reported performance metrics should be interpreted cautiously as preliminary, sample-specific estimates rather than robust indicators of model performance or generalizability.

For comparison with CHAID, we fitted multivariable logistic regression models as conventional regression approaches and LASSO logistic regression models as a penalized alternative to reduce overfitting and improve variable selection. Logistic and LASSO models were used to classify outcome status within the study sample rather than to support causal inference; therefore, the results were interpreted as reflecting association patterns and classification performance rather than causal effects. The same set of candidate predictors used in the CHAID analysis was entered into the logistic and LASSO models to facilitate comparability across analytic approaches. For LASSO logistic regression, the penalty parameter was selected using internal cross-validated deviance within the training set. Classification performance was evaluated separately for each dichotomized outcome—nutritional risk (MNA-SF), impaired SPPB, slow gait speed, and impaired TUG performance—using area under the receiver operating characteristic curve (AUC), sensitivity, specificity, positive predictive value (PPV), negative predictive value (NPV), accuracy, and *F*_1_ score in the test sets. All analyses were conducted using SPSS version 31 (IBM Corp) and R version 4.4.3. No formal subgroup, interaction, nor sensitivity analyses were conducted.

## Results

### Participant Characteristics by Depression Status

A total of 1010 community-dwelling adults were included in the study (Table 1). The median age was 68 (IQR 62.7‐73.0) years, and 757 of 1010 (75%) participants were female. Overall, 143 of 1010 (14.2%) participants screened positive for depressive symptoms, defined as a GDS-15 score ≥5. Because of variable-specific missing data, denominators differed slightly across some analyses.

**Table 1.** Participant characteristics stratified by depression status.

Characteristics	Global sample (n=1010)	GDS-15^a^ classification		Statistical test results (df^b^)	*P* value
		Without depressive symptoms (n=865)	With depressive symptoms (n=143)		
Age (years), median (IQR)	68.0 (62.7‐73.0)	68.0 (63.0‐72.0)	68.0 (61.0‐74.0)	64,473.5^c^	.42
Gender, n (%)				3.42 (1)^d^	.06
Male	253 (25)	226 (26.1)	27 (18.9)		
Female	757 (75)	639 (73.9)	116 (81)		
Education, n (%)				9.42 (2)^d^	.009
Junior high school and below	314 (31.1)	253 (29.2)	60 (42)		
Secondary	303 (30)	265 (30.6)	38 (26.6)		
College and above	393 (38.9)	347 (40.1)	45 (31.5)		
Marital status, n (%)				10.68 (1)^d^	.001
Married, currently living with spouse	697 (69)	614 (71)	82 (57.3)		
Unmarried^e^	313 (31)	251 (29)	61 (42.7)		
Living arrangement, n (%)				21.36 (2)^d^	<.001
Living with spouse only	233 (23.1)	203 (23.5)	29 (20.3)		
Living with family	650 (64.4)	570 (65.9)	79 (55.2)		
Living alone or in institution	127 (12.6)	92 (10.6)	35 (24.5)		
Having religious belief, n (%)	795 (78.8)	670 (77.5)	123 (86)	5.36 (1)^d^	.02
Employment status, n (%)				3.51 (1)^d^	.06
Retired	622 (61.6)	543 (62.8)	78 (54.5)		
Other^f^	388 (38.4)	322 (37.2)	65 (45.5)		
Economic status, n (%)				11.68 (1)^d^	<.001
No financial stress	530 (52.5)	472 (54.6)	56 (39.2)		
Financial stress	480 (47.5)	393 (45.4)	87 (60.8)		
Having an exercise habit (yes), n (%)	834 (82.6)	741 (85.7)	92 (64.3)	38.91 (1)^d^	<.001
Participating in leisure activities (yes), n (%)	636 (63)	557 (64.4)	77 (53.8)	5.85 (1)^d^	.02
Having experienced a fall (yes), n (%)	274 (27.1)	216 (25)	57 (39.9)	14.14 (1)^d^	<.001
Anthropometric measures, median (IQR)					
BMI (kg/m^2^)	24.1 (21.8‐26.5)	24.1 (21.9‐26.6)	23.2 (20.9‐25.6)	54,700.0^c^	.03
Abdominal circumference (cm)	90.0 (83.9‐97.0)	90.0 (84.0‐96.9)	90.0 (83.0‐97.1)	62,351.5^c^	.88
Calf circumference (cm)	35.0 (33.0‐37.0)	35.0 (33.0‐37.4)	34.0 (32.0‐36.2)	50,160.5^c^	<.001
CIRS-G^g^, median (IQR)	6 (3-8)	5 (2-7)	9 (6-12)	149.99 (20)^d^	<.001
CIRS-G SI^h^, median (IQR)	1.2 (1.2‐1.4)	1.2 (1.0‐1.4)	1.4 (1.1‐1.6)	181.28 (46)^d^	<.001

a GDS-15: 15-item Geriatric Depression Scale.

b Degrees of freedom reported for *χ*² tests.

c Mann-Whitney *U* test (Wilcoxon rank-sum test).

d *χ*² test.

e “Unmarried” includes participants who were single, widowed, divorced, or separated.

f “Other” employment status includes participants who were homemakers or currently employed.

g CIRS-G: Cumulative Illness Rating Scale for Geriatrics.

h CIRS-G SI: Cumulative Illness Rating Scale for Geriatrics Severity Index.

Depression status was significantly associated with several sociodemographic characteristics. Educational attainment differed significantly between participants with and without depressive symptoms (*χ*²_2_=9.42, *P*=.009). Specifically, a higher proportion of participants with depressive symptoms had a junior high school education or below (60/143, 42% vs 253/865, 29.2%), whereas a lower proportion had a college education or above (45/143, 31.5% vs 347/865, 40.1%). Marital status, living arrangement, religious belief, and economic status were also significantly associated with depression status. Compared with participants without depressive symptoms, those with depressive symptoms were more likely to be unmarried (61/143, 42.7% vs 251/865, 29%; *χ*²_1_=10.68, *P*=.001), to live alone or in an institution (35/143, 24.5% vs 92/865, 10.6%; *χ*²_2_=21.36, *P*<.001), to report having a religious belief (123/143, 86% vs 670/865, 77.5%; *χ*²_1_=5.36, *P*=.02), and to report financial stress (87/143, 60.8% vs 393/865, 45.4%; *χ*²_1_=11.68, *P*<.001). Employment status did not differ significantly between groups (*χ*²_1_=3.51, *P*=.06).

Regarding lifestyle factors, participants with depressive symptoms were less likely to report regular exercise (92/143, 64.3% vs 741/865, 85.7%; *χ*²_1_=38.91, *P*<.001) and leisure activities (77/143, 53.8% vs 557/865, 64.4%; *χ*²_1_=5.85, *P*=.02) and were more likely to report a history of falls (57/143, 39.9% vs 216/865, 25%; *χ*²_1_=14.14, *P*<.001).

For anthropometric measures, participants with depressive symptoms had a lower median BMI than those without depressive symptoms (23.2, IQR 20.9‐25.6 vs 24.1, IQR 21.9‐26.6 kg/m²; *U*=54,700.0, *P*=.03) and a smaller median calf circumference (34.0, IQR 32.0‐36.2 vs 35.0, IQR 33.0‐37.4 cm; *U*=50,160.5, *P*<.001), whereas abdominal circumference did not differ significantly between groups (90.0, IQR 83.0‐97.1 vs 90.0, IQR 84.0‐96.9 cm; *U*=62,351.5, *P*=.88). Clinical burden also differed significantly by depression status. Participants with depressive symptoms had higher median CIRS-G scores than those without depressive symptoms (9, IQR 6‐12 vs 5, IQR 2‐7; *U*=149,099.0, *P*<.001) and higher median CIRS-G SI scores (1.4, IQR 1.1‐1.6 vs 1.2, IQR 1.0‐1.4; *U*=181,284.0, *P*<.001).

### Associations of Depression Status With Nutritional Risk and Lower-Extremity Physical Performance

As shown in Table 2, the median MNA-SF score was 13 (IQR 12‐14), and 159 of 1010 (15.8%) participants were classified as at risk of malnutrition or malnourished. Participants with depressive symptoms had poorer nutritional status than those without depressive symptoms, as indicated by lower median MNA-SF scores (12, IQR 11‐14 vs 14, IQR 12‐14; *U*=40,275.0, *P*<.001) and a higher proportion classified as at risk of malnutrition or malnourished (53/143, 37.1% vs 105/867, 12.1%; *χ*²_1_=57.67, *P*<.001).

For lower-extremity physical performance, the median SPPB score was 11 (IQR 10‐12), and 172 of 1010 (17%) participants met the criteria for impairment. Compared with participants without depressive symptoms, those with depressive symptoms had lower median SPPB scores (10, IQR 9‐12 vs 12, IQR 10‐12; *U*=39,722.0, *P*<.001) and were more likely to be classified as impaired (53/143, 37.1% vs 118/867, 13.6%; *χ*²_1_=50.48, *P*<.001). The median gait speed was 1.1 m/s, and 343 of 1010 (34%) participants were classified as impaired. Participants with depressive symptoms had slower median gait speed than those without depressive symptoms (0.9, IQR 0.8‐1.2 vs 1.1, IQR 0.9‐1.3 m/s; *U*=41,132.0, *P*<.001) and were more likely to have impaired gait speed (73/143, 51% vs 270/865, 31.2%; *χ*²_1_=51.41, *P*<.001). The median TUG time was 8.2 seconds, and 80 of 1010 (7.9%) participants met the impairment threshold. Participants with depressive symptoms had longer median TUG times than those without depressive symptoms (9.3, IQR 7.8‐11.4 vs 8.1, IQR 7.0‐9.5 seconds; *U*=78,609.0, *P*<.001) and were more likely to be classified as impaired (25/143, 17.5% vs 55/864, 6.4%; *χ*²_1_=22.02, *P*<.001). Overall, depressive symptoms were consistently associated with poorer nutritional status and lower-extremity physical performance.

**Table 2.** Nutritional risk and lower-extremity physical performance by depression status.

Nutritional risk and lower-extremity physical performance	Global sample	GDS-15^a^ classification		Statistical test results (df^b^)	*P* value
		Without depressive symptoms	With depressive symptoms		
MNA-SF^c^ score, median (IQR)	13 (12‐14)	14 (12‐14)	12 (11‐14)	40,275^d^	<.001
MNA-SF classification, n (%)				57.67 (1)^e^	<.001
At risk or malnourished	159 (15.8)	105 (12.1)	53 (37.1)		
Well-nourished	851 (84.3)	760 (87.9)	90 (62.9)		
Lower-extremity physical performance					
SPPB^f^ score, median (IQR)	11 (10‐12)	12 (10‐12)	10 (9‐12)	39,722^d^	<.001
SPPB classification, n (%)				50.48 (1)^e^	<.001
Impaired^g^	172 (17)	118 (13.6)	53 (37.1)		
Normal mobility	831 (82.3)	744 (86)	86 (60.1)		
Gait speed, median (IQR)					
6-meter walking speed (m/s)	1.1 (0.9‐1.3)	1.1 (0.9‐1.3)	0.9 (0.8‐1.2)	41,132^d^	<.001
Gait speed classification, n (%)				51.41 (1)^e^	<.001
Impaired^g^	343 (34)	270 (31.2)	73 (51)		
Normal	665 (65.8)	594 (68.8)	70 (49)		
TUG^h^ (s), median (IQR)	8.2 (7.1‐9.8)	8.1 (7.0‐9.5)	9.3 (7.8‐11.4)	78,609^d^	<.001
TUG classification, n (%)				22.02 (1)^e^	<.001
Impaired^g^	80 (7.9)	55 (6.4)	25 (17.5)		
Normal	924 (91.5)	809 (93.5)	114 (79.7)		

a GDS-15: 15-item Geriatric Depression Scale.

b Degrees of freedom reported for *χ*² tests.

c MNA-SF: Mini Nutritional Assessment–Short Form.

d Mann-Whitney *U* test (Wilcoxon rank-sum test).

e *χ*² test.

f SPPB: Short Physical Performance Battery.

g “Impaired” indicates the proportion of participants who met the cutoff for impairment: MNA-SF≤11; SPPB≤9; gait speed of 6-meter walk test≤1.0 m/s; timed up-and-go (TUG) test≥12 seconds.

h TUG: timed up-and-go.

### CHAID Decision Tree Analysis for Nutritional Status (MNA-SF)

**Figure 1.**
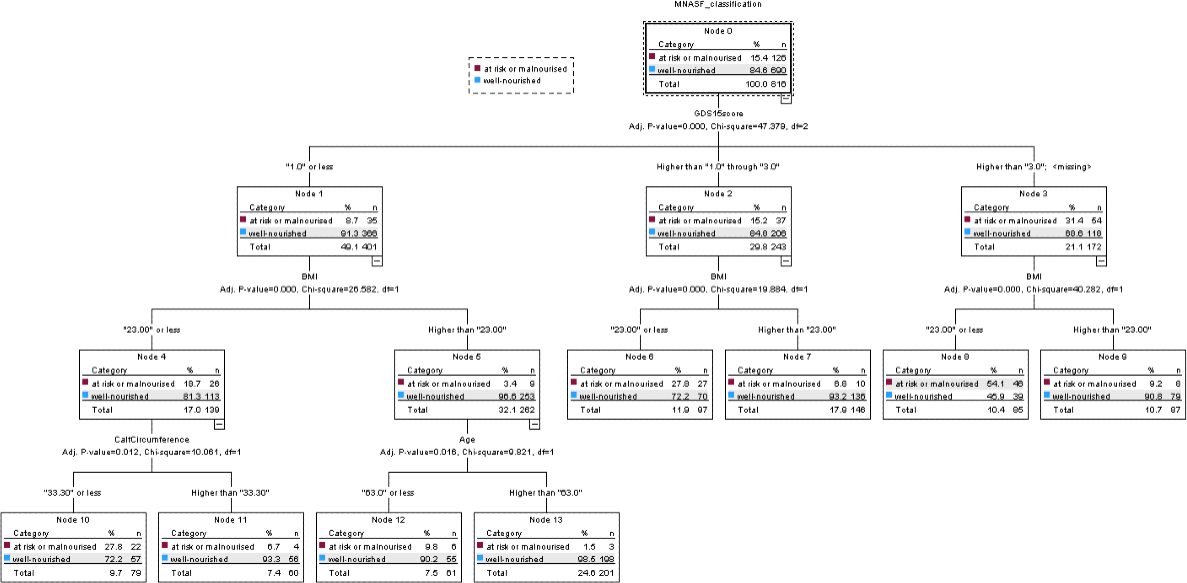
Chi-square automatic interaction detection (CHAID) decision tree for nutritional status classified using the Mini Nutritional Assessment–Short Form (MNA-SF). Terminal nodes show the number and proportion of participants classified as well-nourished or at risk of malnutrition or malnourished. For improved readability, a higher-resolution version of this figure is reproduced in Multimedia Appendix 1.

The CHAID decision tree model for nutritional status based on the MNA-SF is presented in Figure 1. The MNA-SF ranges from 0 to 14, with higher scores indicating better nutritional status and scores ≤11 indicating risk of malnutrition or malnutrition. Nutritional status was modeled as a binary outcome classified as well-nourished versus at risk of malnutrition or malnourished.

Depressive symptom severity, measured using the GDS-15, was identified as the primary splitting variable in the decision tree (*P*<.001). The GDS-15 ranges from 0 to 15, with higher scores indicating greater depressive symptom severity and scores ≥5 indicating clinically relevant depressive symptoms. The model divided participants into 3 depressive symptom groups (≤1, 2‐3, and ≥4), corresponding to different levels of nutritional vulnerability within the study sample.

Within each depressive symptom stratum, BMI served as the main secondary splitting variable, indicating that anthropometric status further differentiated nutritional status patterns beyond depressive symptom severity alone. Additional hierarchical refinement was provided by calf circumference and age, which identified subgroups with distinct nutritional vulnerability profiles. Smaller calf circumference, a proxy indicator of reduced muscle mass, and older age were associated with a higher prevalence of nutritional risk within specific depressive symptoms and BMI strata.

Participants with GDS-15 scores ≤1 were predominantly well-nourished, with 91.3% (366/401) classified as having normal nutritional status. However, nutritional risk was higher among individuals with lower BMI and smaller calf circumference, with 27.8% (22/79) classified as at risk of malnutrition or malnourished within this otherwise low depressive symptom group. Nutritional vulnerability was substantially greater among participants with higher depressive symptom severity. In particular, among participants with GDS-15 scores ≥4 and BMI≤23.0 kg/m², 54.1% (45/85) were classified as at risk of malnutrition or malnourished, representing the subgroup with the highest prevalence of nutritional risk identified by the model.

Each terminal node of the decision tree represents a distinct subgroup defined by combinations of depressive symptoms, BMI, calf circumference, and age and displays the corresponding number and proportion of participants classified according to nutritional status. This hierarchical structure illustrates how depressive symptoms together with anthropometric and demographic factors differentiate subgroups with varying prevalences of nutritional risk among community-dwelling adults aged ≥50 years. In addition, the CHAID model had acceptable within-sample classification performance, with accuracies of 84.4% in the training dataset and 85.6% in the test dataset.

### CHAID Decision Tree Analysis for Lower Extremity Physical Performance

#### Short Physical Performance Battery (SPPB)

**Figure 2.**
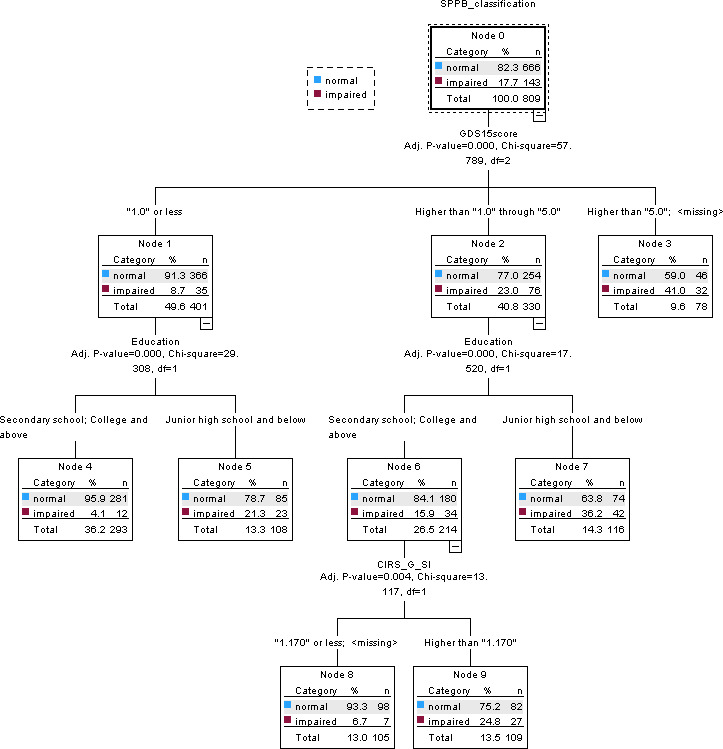
Chi-square automatic interaction detection (CHAID) decision tree for lower-extremity physical performance classified using the Short Physical Performance Battery (SPPB). Terminal nodes show the number and proportion of participants classified as normal or impaired. CIRS-G SI: Cumulative Illness Rating Scale for Geriatrics Severity Index; GDS-15: 15-item Geriatric Depression Scale.

The CHAID decision tree model for lower-extremity physical performance based on the SPPB is presented in Figure 2. The SPPB ranges from 0 to 12, with higher scores indicating better physical performance and scores ≤9 indicating impaired lower-extremity function. An improvement of approximately 1 point has been suggested as a clinically meaningful change in community-dwelling older adults.

Depressive symptom severity, measured using the GDS-15, was identified as the primary splitting variable in the decision tree (*P*<.001), dividing participants into 3 groups (≤1, >1 to ≤5, and >5). These findings suggest that depressive symptom burden was an important factor in differentiating patterns of lower-extremity functional impairment.

Among participants with lower depressive symptom scores (≤1 and >1 to ≤5), education level emerged as the most influential secondary predictor. Impairment prevalence was substantially higher among individuals with a junior high school education or below than among those with at least secondary education. Specifically, impairment prevalence was 21.3% (23/108) versus 4.1% (12/293) in the ≤1 group and 36.2% (42/116) versus 15.9% (34/214) in the >1 to ≤5 group. These results suggest that lower educational attainment was associated with a higher prevalence of functional impairment, even among individuals with relatively low depressive symptom burden.

Within the intermediate depressive symptom group (>1 to ≤5), comorbidity severity, assessed using the CIRS-G SI, provided further hierarchical refinement. Participants with higher comorbidity severity had a higher impairment prevalence, reaching 24.8% (27/109), suggesting that comorbidity burden may further distinguish impairment patterns beyond depressive symptoms and education level. Participants with GDS-15 scores >5 exhibited consistently elevated impairment prevalence (32/78, 41%), regardless of education level or comorbidity severity. This finding suggests that higher depressive symptom severity was associated with impaired lower-extremity physical performance in this sample.

Each terminal node represents a distinct subgroup defined by combinations of depressive symptom severity, education level, and comorbidity burden and displays the corresponding number and proportion of participants classified according to SPPB impairment status. This hierarchical structure illustrates how depressive symptoms, together with sociodemographic and clinical factors, differentiate subgroups with varying prevalences of functional impairment among community-dwelling adults aged ≥50 years. In addition, the CHAID model had acceptable within-sample classification performance, with accuracies of 82.3% in the training dataset and 85.1% in the test dataset.

#### Gait Speed

**Figure 3.**
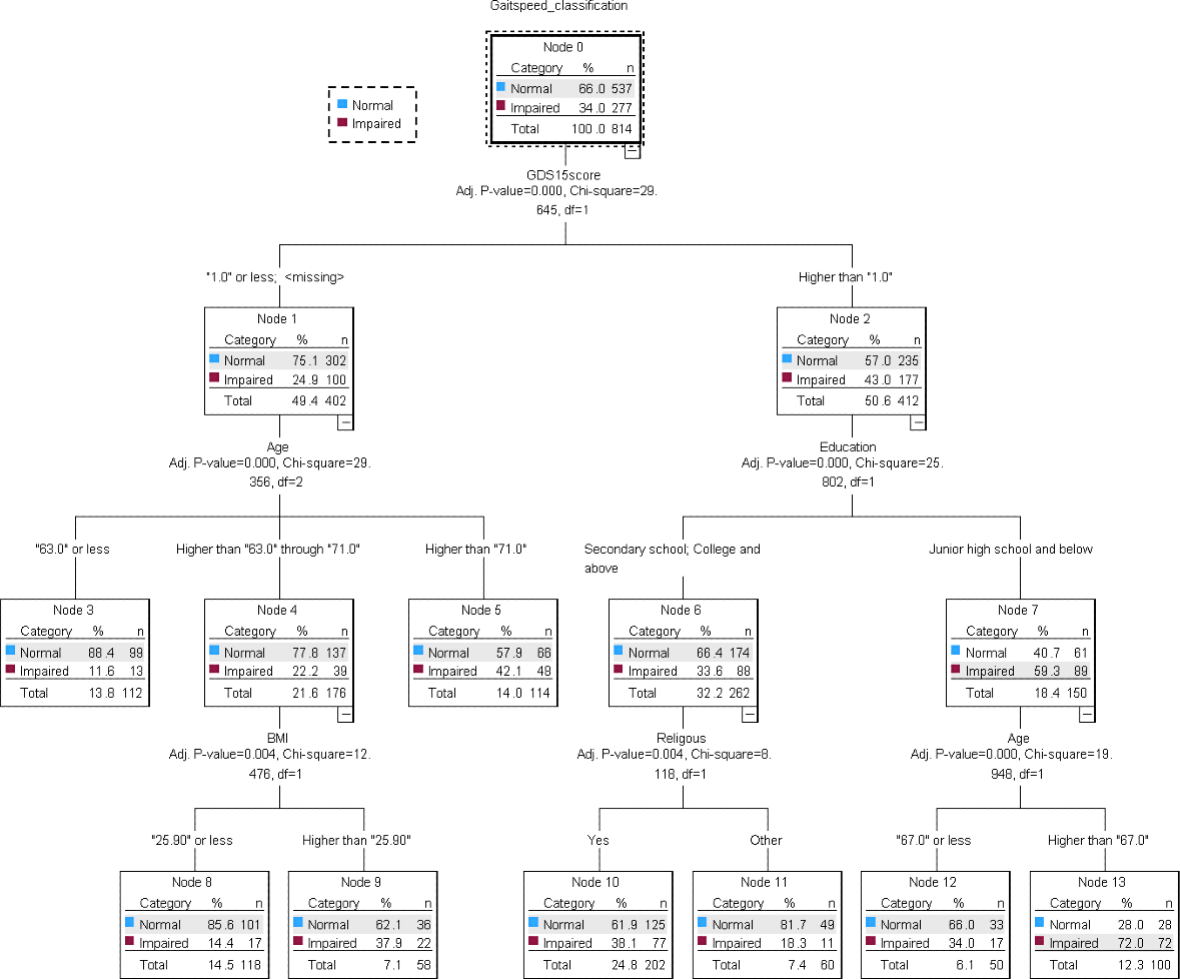
Chi-square automatic interaction detection (CHAID) decision tree for lower-extremity physical performance classified using gait speed. Terminal nodes show the number and proportion of participants classified as normal or impaired. GDS-15: 15-item Geriatric Depression Scale.

The CHAID decision tree model for gait speed impairment is presented in Figure 3. Gait speed impairment was defined using a cutoff of ≤1.0 m/s, consistent with established thresholds for identifying mobility limitation in older adults. Habitual gait speed is a clinically meaningful indicator of functional status, and changes of approximately 0.05 m/s to 0.10 m/s have been associated with meaningful functional differences in community-dwelling populations.

Depressive symptom severity, measured using the GDS-15, was identified as the primary splitting variable in the decision tree (*P*<.001), dividing participants into 2 groups (≤1 and >1). This finding suggests that depressive symptom burden was an important factor in differentiating patterns of gait speed impairment.

Among participants with lower depressive symptom scores (GDS-15≤1), gait speed impairment was further differentiated by age and BMI. Impairment prevalence increased substantially with advancing age, ranging from 11.6% (13/112) among participants aged ≤63 years to 42.1% (48/114) among those aged >71 years. Within the intermediate age group (63-71 years), BMI provided additional hierarchical refinement. Impairment prevalence increased from 14.4% (17/118) among participants with BMI≤25.9 kg/m² to 37.9% (22/58) among those with BMI>25.9 kg/m², suggesting that higher BMI was associated with a higher prevalence of gait speed impairment within this age group.

Among participants with higher depressive symptom scores (GDS-15>1), education level emerged as the most influential secondary predictor. Impairment prevalence was substantially higher among individuals with a junior high school education or below (89/150, 59.3%) than among those with higher educational attainment (88/262, 33.6%), suggesting that lower educational attainment was associated with a higher prevalence of gait speed impairment. Religious affiliation and older age (>67 years) provided additional hierarchical refinement, identifying subgroups with further elevated impairment prevalence.

Each terminal node represents a distinct subgroup defined by combinations of depressive symptom severity, age, BMI, education level, and religious affiliation and displays the corresponding number and proportion of participants classified according to gait speed impairment status. This hierarchical structure illustrates how depressive symptoms, together with demographic, anthropometric, and social factors, differentiate subgroups with varying prevalences of gait speed impairment among community-dwelling adults aged ≥50 years. In addition, the CHAID model had acceptable within-sample classification performance, with accuracies of 71.4% in the training dataset and 75.3% in the test dataset.

#### Timed Up-and-Go (TUG)

**Figure 4.**
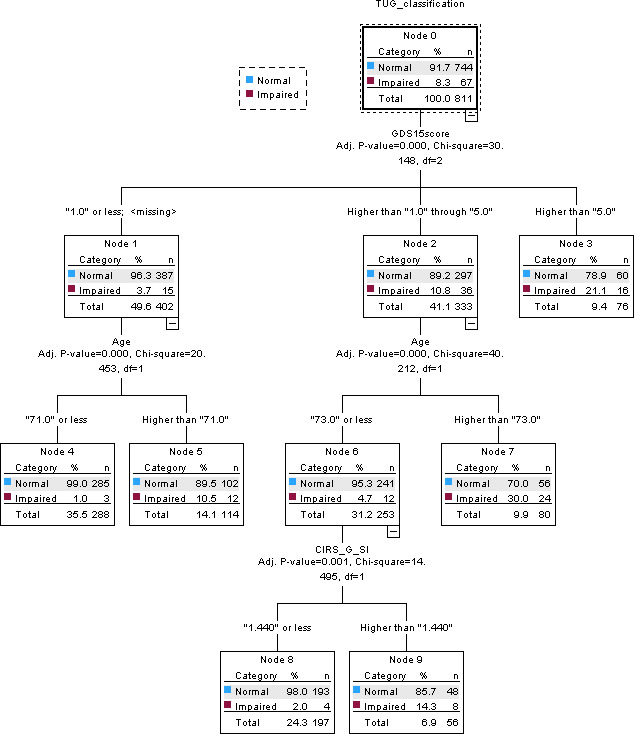
Chi-square automatic interaction detection (CHAID) decision tree for lower-extremity physical performance classified using the timed up-and-go (TUG) test. Terminal nodes show the number and proportion of participants classified as normal or impaired. CIRS-G SI: Cumulative Illness Rating Scale for Geriatrics Severity Index; GDS-15: 15-item Geriatric Depression Scale.

The CHAID decision tree model for TUG impairment is presented in Figure 4. TUG performance was assessed using the standard 3-meter test, with longer completion times indicating poorer mobility performance. Impairment was defined as a completion time of ≥12 seconds, consistent with established thresholds for identifying mobility limitation in community-dwelling older adults. Although clinically meaningful change thresholds vary across populations, reported minimal clinically important differences for TUG typically range from approximately 1.2 seconds to 3.4 seconds.

Depressive symptom severity, measured using the GDS-15, was identified as the primary splitting variable in the decision tree (*P*<.001), dividing participants into 3 groups (≤1, >1 to ≤5, and >5). Participants with GDS-15 scores ≤1 had the lowest prevalence of TUG impairment (15/402, 3.7%). However, impairment prevalence increased with advancing age, reaching 10.5% among participants aged >71 years, suggesting that age further differentiated TUG impairment patterns even among individuals with minimal depressive symptoms.

Within the intermediate depressive symptom group (GDS-15 >1 to ≤5), age served as the most influential secondary predictor. Impairment prevalence increased substantially among participants aged >73 years, reaching 30% (24/80), suggesting that depressive symptom burden together with advanced age was associated with a higher prevalence of TUG impairment. Comorbidity severity, assessed using the CIRS-G SI, provided additional hierarchical refinement. Participants with higher comorbidity severity (CIRS-G SI >1.44) had a higher prevalence of impairment, reaching 14.3% (8/56) among younger individuals, suggesting that comorbidity burden may further distinguish TUG impairment patterns beyond depressive symptoms and age. Participants with GDS-15 scores >5 exhibited consistently an elevated prevalence of impairment (16/76, 21.1%), suggesting that higher depressive symptom severity was strongly associated with impaired functional mobility in this sample.

Each terminal node represents a distinct subgroup defined by combinations of depressive symptom severity, age, and comorbidity burden and displays the corresponding number and proportion of participants classified according to TUG impairment status. This hierarchical structure illustrates how depressive symptoms, together with clinical and demographic factors, differentiate subgroups with varying prevalences of TUG impairment among community-dwelling adults aged ≥50 years. In addition, the CHAID model had acceptable within-sample classification performance, with accuracies of 91.7% in the training dataset and 93.3% in the test dataset.

### Summary of CHAID Model Findings

Across all models, the GDS-15 score consistently emerged as the primary splitting variable. Secondary predictors, including BMI, calf circumference, age, comorbidity severity, and education level, provided additional hierarchical refinement and helped differentiate subgroups with varying prevalences of nutritional risk and lower-extremity functional impairment. Overall, these findings suggest that CHAID may serve as a useful exploratory approach for characterizing within-sample patterns, although the performance estimates should be interpreted as preliminary and sample-specific.

### Comparison of Interpretable Machine Learning and Regression Models

Table 3 summarizes the classification performance of CHAID, logistic regression, and LASSO logistic regression models for nutritional status (MNA-SF) and lower-extremity physical performance outcomes, including SPPB, gait speed, and TUG.

**Table 3.** Classification performance for nutritional status and lower-extremity physical performance across chi-square automatic interaction detection (CHAID), logistic regression, and least absolute shrinkage and selection operator (LASSO) models.

Outcome and model		AUC^a^ (95% CI)	Sensitivity, %	Specificity, %	PPV^b^	NPV^c^	Accuracy, %	*F*_1_ score
MNA-SF^d^								
CHAID model		0.80 (0.72‐0.88)	57.6	88.8	0.91	0.51	83.5	90
Logistic regression model		0.85 (0.79‐0.92)	83.9	69.7	1.00	0.00	63.6	77.8
Logistic regression LASSO model		0.96 (0.92‐0.99)	98.8	78.8	0.96	0.93	95.4	97.2
SPPB^e^								
CHAID model		0.79 (0.71‐0.87)	0	100	N/A^f^	0.85	0.85	N/A
Logistic regression model		0.87 (0.82‐0.94)	86.2	82.4	1.00	0.00	3	5.9
Logistic regression LASSO model		0.93 (0.88‐0.97)	89.7	90.3	0.62	0.98	90.2	73.2
Gait speed								
CHAID model		0.77 (0.70‐0.83)	56.1	86.7	0.69	0.79	76.3	61.7
Logistic regression model		0.92 (0.87‐0.96)	83.3	89.1	1.00	0.71	73.2	35
Logistic regression LASSO model		0.95 (0.92‐0.98)	86.4	94.5	0.89	0.93	91.8	87.7
TUG^g^								
CHAID model		0.74 (0.71‐0.78)	100	48.9	0.12	1.00	52.3	22
Logistic regression model		1.00 (1-1)	21.2	100	1.00	0.00	4.4	8.5
Logistic regression LASSO model		0.5 (0.5‐0.5)	0	100	NA	0.93	93.3	N/A

a AUC: area under the curve.

b PPV: positive predictive value.

c NPV: negative predictive value.

d MNA-SF: Mini Nutritional Assessment–Short Form.

e SPPB: Short Physical Performance Battery.

f N/A: not applicable.

g TUG: timed up-and-go.

For nutritional status assessed using MNA-SF, the CHAID model had acceptable discrimination, with an AUC of 0.80 (95% CI 0.72‐0.88), characterized by high specificity but lower sensitivity. Logistic regression analysis had higher overall discrimination, achieving an AUC of 0.85 (95% CI 0.79‐0.92), with increased sensitivity but reduced specificity compared with CHAID. The LASSO logistic regression model had the highest classification performance, with an AUC of 0.96 (95% CI 0.92‐0.99), together with the highest overall accuracy and *F*_1_ score.

For lower-extremity physical performance outcomes, model performance varied by outcome measure. For SPPB impairment, all models had acceptable discrimination, with AUC values of 0.79 for the CHAID model, 0.87 for the logistic regression model, and 0.93 for the LASSO logistic regression model. The CHAID model had high specificity but low sensitivity, indicating a lower ability to identify impaired cases. Logistic regression and LASSO logistic regression models had more balanced sensitivity and specificity profiles, with LASSO yielding the highest overall discrimination and classification accuracy. For gait speed impairment, the CHAID model had moderate discrimination, with an AUC of 0.77 (95% CI 0.70‐0.83), characterized by lower sensitivity and higher specificity. The logistic regression and LASSO logistic regression models had higher discrimination, with AUC values of 0.92 (95% CI 0.87‐0.96) and 0.95 (95% CI 0.92‐0.98), respectively. The LASSO model also achieved the highest overall classification accuracy and *F*_1_ score among the 3 approaches. For TUG impairment, model performance was less consistent. The CHAID model had fair discrimination, with an AUC of 0.74 (95% CI 0.71‐0.78), characterized by relatively high sensitivity but lower specificity and overall accuracy. The logistic regression model achieved an AUC of 1.00; however, the accompanying classification metrics suggested possible overfitting, as indicated by low sensitivity and overall accuracy despite perfect specificity. The LASSO logistic regression model had poor discrimination, with an AUC of 0.50, indicating limited usefulness for characterizing this outcome in the current sample.

Overall, the LASSO logistic regression model had the strongest and most consistent classification performance for MNA-SF, SPPB, and gait speed outcomes in this sample. In contrast, the CHAID model generally had lower sensitivity but offered transparent hierarchical subgroup characterization. Model performance for TUG was less consistent across approaches, suggesting that this outcome may be more difficult to characterize using the current set of predictors.

## Discussion

### Principal Findings

In this cross-sectional secondary analysis, depressive symptoms were associated with poorer nutritional status and lower-extremity physical performance among middle- to older-aged adults. Although both the tree-based exploratory approach and regression models identified broadly similar association patterns, the CHAID model offered a more transparent representation of subgroup structure within the study sample. Using data from a large community-based sample, this study examined the cross-sectional associations of depressive symptom severity with nutritional status and lower-extremity physical performance among community-dwelling adults aged 50 years and older. The findings suggest that greater depressive symptom severity was associated with greater nutritional vulnerability and poorer mobility-related performance. These results are consistent with prior studies linking depressive symptoms to frailty-related physiological and functional decline [10,13,17,19].

Using CHAID decision tree analysis, this study identified a hierarchical subgroup structure in which depressive symptom severity was consistently selected as the primary splitting variable across 2 outcome domains: nutritional status and lower-extremity physical performance. Additional predictors, including age, BMI, calf circumference, education level, and comorbidity severity, further refined subgroup patterns across these outcomes. Rather than serving as a definitive risk stratification tool, CHAID in this study provided rule-based subgroup characterization, with potential advantages for visualizing and communicating clinically relevant patterns within the sample. Together, these findings suggest that depressive symptoms may represent an important clinical marker within the multidimensional pathways linking psychological, nutritional, and functional health in aging populations. Importantly, our findings support the view that health status may be conceptualized as a continuous spectrum, such that older adults classified as low risk or as having only mild impairment may still exhibit substantial internal heterogeneity [46,47] and variation in scores below the conventional cutoff may still carry clinical warning value and help distinguish subgroups with different levels of nutritional vulnerability and functional impairment, which is particularly meaningful for prevention-oriented assessment.

In the comparative model analyses, the LASSO logistic regression model had the highest classification performance and the most balanced sensitivity and specificity profiles for the MNA-SF, SPPB, and gait speed outcomes, suggesting that regularized regression analysis may be useful for classification in datasets with multiple correlated clinical variables. In contrast, although the CHAID decision tree model generally had lower sensitivity, it demonstrated acceptable within-sample classification performance and offered a transparent, rule-based approach for subgroup characterization. Rather than serving as a definitive screening or stratification tool, CHAID may provide complementary value by making subgroup patterns, threshold splits, and variable interactions easier to visualize and communicate in clinical contexts. Taken together, these findings suggest complementary roles for the tree-based exploratory approach and regression-based methods: The LASSO regression model had higher classification performance, whereas the CHAID model offered transparent, rule-based subgroup characterization that may facilitate visualization and communication of clinically relevant patterns within the study sample.

### Comparison With Previous Work

These findings extend prior research on multifactorial geriatric assessment by showing that routinely collected clinical and functional measures can be examined together using a transparent, interpretable analytic approach to characterize patterns of nutritional vulnerability and mobility impairment. Consistent with previous studies emphasizing the multidimensional nature of frailty and functional decline [1,36,48,49], the CHAID decision tree model identified a hierarchical subgroup structure that differentiated participants with varying levels of nutritional and physical vulnerability. More broadly, these findings illustrate how interpretable analytic approaches may complement conventional geriatric assessment by supporting transparent pattern recognition and subgroup characterization.

Overall, 14.2% of participants in our study screened positive for depressive symptoms, which was lower than the 21.3% prevalence reported in a previous study [5]. This difference may reflect variation in sample characteristics, recruitment settings, and population composition across studies. Compared with those without depressive symptoms, participants with depressive symptoms had poorer nutritional status and lower-extremity physical performance, including higher malnutrition risk, lower SPPB scores, and slower gait speed. These associations are consistent with established physiological and behavioral mechanisms linking depression to frailty progression, as depression has been associated with reduced motivation, appetite, and physical activity, which may contribute to reduced dietary intake, sarcopenia, and functional decline [8,10-14]. Taken together, these findings support the relevance of depressive symptoms as a clinical marker of multidimensional vulnerability in aging populations. The decision tree analysis further extends previous work by illustrating how depressive symptoms interact with demographic, anthropometric, and clinical factors to differentiate subgroups with varying levels of nutritional and functional vulnerability. Nutritional and functional patterns varied according to depressive symptom severity in combination with body composition, age, education level, and comorbidity burden. These hierarchical subgroup patterns align with prior evidence linking depression, sarcopenia, and functional decline while also offering a transparent way to visualize and describe heterogeneity within the sample.

At the health systems level, these findings are consistent with international recommendations advocating integrated care models that address mental health, nutrition, and physical function as interconnected determinants of healthy aging [48,49]. Incorporating depression screening into routine geriatric assessment may help identify older adults with coexisting nutritional and functional vulnerabilities. Interpretable analytic approaches such as CHAID may support this process by providing transparent, rule-based subgroup characterization. However, external validation and prospective evaluation are still needed before such approaches can be considered for broader clinical use. In addition, this study focused on a selected tree-based exploratory approach and did not compare the full range of interpretable machine learning methods.

### Strengths and Limitations

This study has several important strengths. First, it applied both a tree-based exploratory approach and regression-based approaches to examine depression-related patterns using routinely collected geriatric assessment measures. This comparative framework enabled assessment of both classification performance and subgroup interpretability. Second, the use of well-validated clinical instruments, including the GDS-15, MNA-SF, SPPB, gait speed, and TUG, enhances the clinical relevance of the findings. Third, the relatively large community-based sample supported model estimation and subgroup characterization across nutritional and lower-extremity functional outcomes. Finally, the tree-based exploratory approach enabled transparent, rule-based identification of hierarchical subgroup patterns, which may facilitate visualization and communication of clinically relevant heterogeneity within the sample.

Several limitations should also be considered. First, the study focused on a selected tree-based exploratory approach and did not compare the full range of interpretable machine learning methods. Second, model evaluation relied only on an internal training-testing split, without cross-validation, bootstrapping, or external validation. Accordingly, the reported performance metrics should be interpreted as exploratory and sample-specific and should not be taken as evidence of generalizability beyond the current sample. Future studies should incorporate more rigorous internal validation procedures and external validation in independent populations. Third, the cross-sectional study design precludes causal inference and does not establish temporal relationships among depressive symptoms, nutritional status, and physical performance. Fourth, participants were recruited through convenience sampling from a single geographic region, which may limit generalizability to other populations or health care settings. Fifth, depressive symptoms were assessed using a screening instrument rather than a diagnostic interview and therefore may not fully capture clinical depression severity. Finally, model performance varied across outcomes, particularly for TUG impairment, suggesting that additional predictors or alternative analytic approaches may be needed to better characterize this outcome.

### Conclusions

In this community-based sample of adults aged 50 years and older, depressive symptom severity was associated with nutritional vulnerability and poorer lower-extremity physical performance. Using a CHAID decision tree approach, this study identified hierarchical subgroup patterns showing that depressive symptoms, including subthreshold levels, together with age, BMI, calf circumference, education level, and comorbidity burden, were related to variation in nutritional and functional vulnerability. Although LASSO logistic regression analysis had higher overall classification performance, the CHAID model offered transparent, rule-based subgroup characterization that may facilitate visualization and communication of clinically relevant patterns within the study sample. These findings suggest that combining depression screening with nutritional and functional assessment may help identify older adults with coexisting vulnerabilities and may support prevention-oriented geriatric assessment. They also support the view that health status may be better understood as a continuum, such that even depressive symptom scores below the conventional cutoff may still carry clinical relevance.

## Supplementary material

10.2196/94510 Multimedia Appendix 1 Enlarged version of Figure 1 showing a Chi-square automatic interaction detection (CHAID) decision tree for nutritional status classified using the Mini Nutritional Assessment–Short Form (MNA-SF).

## Abbreviations

AUC: area under the curve
CHAID: chi-square automatic interaction detection
CIRS-G: Cumulative Illness Rating Scale for Geriatrics
CIRS-G SI: Cumulative Illness Rating Scale for Geriatrics Severity Index
GDS-15: 15-item Geriatric Depression Scale
LASSO: least absolute shrinkage and selection operator
MNA-SF: Mini Nutritional Assessment–Short Form
NPV: negative predictive value
PPV: positive predictive value
SPPB: Short Physical Performance Battery
STROBE: Strengthening the Reporting of Observational Studies in Epidemiology
TUG: timed up-and-go
