# Supplementary figures and images for: Depression-Related Nutritional Risk and Physical Performance in Middle- to Older-Aged Adults: Cross-Sectional Secondary Analysis of Tree-Based and Regression Approaches
Source: JMIR Med Inform. 2026 Jun 1;14:e94510. doi: 10.2196/94510 (PMC13225840; doi:10.2196/94510)

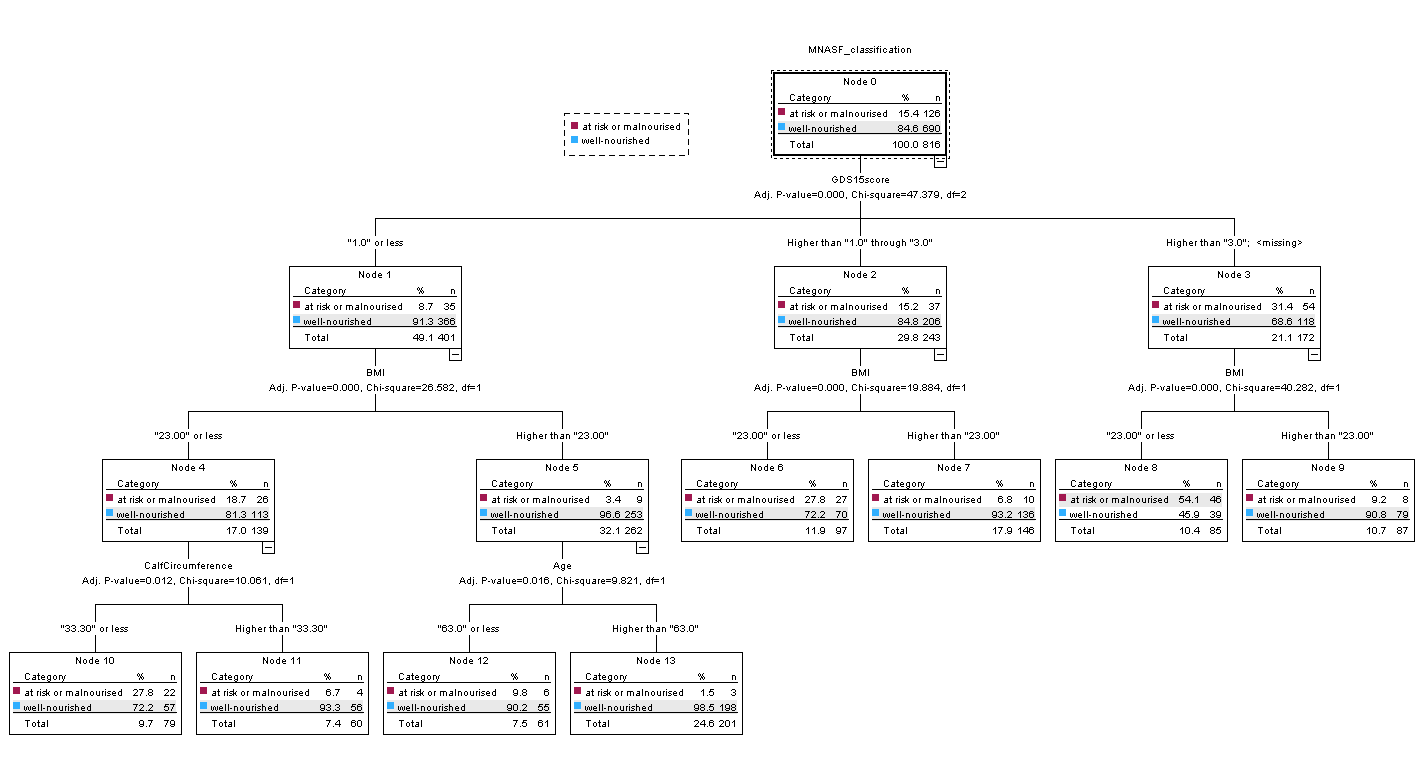

Supplement: Multimedia Appendix 1 [file medinform-v14-e94510-s001.png]
